# Darwinism vs. Medicine

**Published:** 1875-01

**Authors:** 


Darwinism vs. Medicine.—The Paris correspondent of the *Daily Graphic* writes: "I was lately conversing with M. Louis Langue, one of the most ardent of the few French admirers of M. Darwin, and during the interview M. Langue developed a a very remarkable consequence of the doctrine of the survival of the fittest. He urged that inasmuch as species and individuals survive in the struggle for life only by reason of their superior ability to master their environment, whereby it comes to pass that the strong propagate the strong and perfection is reached, it is unscientific and unjustifiable to interfere with nature in her work in any way whereby those who are unfit to survive shall be aided in their struggle. Man is not exempt from the action of the general law of the survival of the fittest; and, therefore, any means whereby the weak are kept in existence, and it is rendered possible for them to propagate their species, is indefensible. When the Spartans killed their deformed offspring they were aiding nature in the accomplishment of its designs; but much more shall we aid in this direction if we shall entirely do away with doctors, medicines, hospitals and all the adjuncts of the healing art. The world is. overpopulated. There was a time when it was possible for plagues and epidemics to kill off the weaker—the surplusage—of humanity, and leave on the earth only those who, by reason of their high physical condition, were able to survive, but the inventions by which we have learned to cuddle the weak, to doctor and bolster them against nature, so that now consumptives are legion, and there is hardly a man without some latent disease, have brought about a condition of affairs which it is absolutely necessary to change if the race is to improve. Let those who are weak go down, as nature intended they should, when attacked by disease, and in a few generations the descendants of the strong alone will survive. M. Langue is so steady a devotee of this idea that when his own child was ill a few months ago he refused to call in a physician, and the child died. Whether his practice will be generally accepted is very questionable, even though one sees little fault to find with his theory.—*Phar. Gazette.*

